# Granule regulation by phase separation during *Drosophila* oogenesis

**DOI:** 10.1042/ETLS20190155

**Published:** 2020-06-23

**Authors:** M. Sankaranarayanan, Timothy T. Weil

**Affiliations:** Department of Zoology, University of Cambridge, Downing Street, Cambridge CB2 3EJ, U.K.

**Keywords:** developmental biology, phase separation, RNP granules

## Abstract

*Drosophila* eggs are highly polarised cells that use RNA–protein complexes to regulate storage and translational control of maternal RNAs. Ribonucleoprotein granules are a class of biological condensates that form predominantly by intracellular phase separation. Despite extensive *in vitro* studies testing the physical principles regulating condensates, how phase separation translates to biological function remains largely unanswered. In this perspective, we discuss granules in *Drosophila* oogenesis as a model system for investigating the physiological role of phase separation. We review key maternal granules and their properties while highlighting ribonucleoprotein phase separation behaviours observed during development. Finally, we discuss how concepts and models from liquid–liquid phase separation could be used to test mechanisms underlying granule assembly, regulation and function in *Drosophila* oogenesis.

## Introduction

Intracellular localisation of messenger RNAs (mRNAs) is a conserved mechanism for achieving compartmentalised protein expression in polarised cells such as neurons and fibroblasts [1–5]. To generate precise protein synthesis and prevent ectopic expression, mRNA localisation is often coupled to translational regulation. One way to achieve this regulation is through binding of trans-acting RNA binding proteins (RBP) to *cis*-acting sequences in the mRNA, which together form micron-sized compartments called ribonucleoprotein (RNP) granules [6–10]. These granules function in the packaging, transport and translational control of mRNAs, and can rapidly respond to cellular and external stimuli. With the ability to control spatial and temporal gene expression, understanding how RNP granules form and disassemble is a key question in cell biology.

RNP granules belong to a class of organelles which lack a physical membrane that separate their contents from the cytoplasm. Different from the commonly known membrane-bound organelles such as the endoplasmic reticulum and Golgi apparatus, membrane-less granules (also referred to as biomolecular condensates) constitute an additional level of macromolecular organization in the cell [11–13]. Most commonly forming via liquid–liquid phase separation (LLPS), these condensates function as microenvironments for cellular reactions [14–16]. This process of ‘de-mixing’ allows RNA and protein molecules to condense into a concentration dependent dense phase which coexists with the soluble cytoplasmic phase [17–20]. The idea that cellular contents exhibit liquid-like characteristics was proposed by multiple groups over the past century, but received renewed interest when P granules in the *C. elegans* embryo were shown to exhibit liquid-like behaviour [21,22]. This discovery has since led to a dramatic increase in the research of biomolecular condensates [18,23–35]. For a more extensive discussion on the physics of condensates, we refer readers to several excellent reviews [36–42].

Our current understanding of the physicochemical principles regulating condensates has been primarily elucidated through *in vitro* studies of purified RNP components under idealised conditions [43]. This approach has been instrumental in describing the role of non-equilibrium features of living cells including post-translational modifications and ATP driven processes, in addition to identifying sequence and structural determinants that control condensate phase behaviour [14,23,44–49]. However, why cells need these compartments, when are they utilised, and what their biochemical and biological functions are remain largely unanswered.

RNP granules, which typically form in response to accumulation of RNAs, are abundant in diverse oocytes including *C. elegans*, *Drosophila* and *Xenopus* [50–53]. Oocytes are highly specialised cells which often rely on RNP granules to localise maternal transcripts for pattern formation in the early embryo [5,54–56]. Oocytes therefore offer a unique opportunity to test the physicochemical principles of phase separation in a living system and further explore the biological role of RNP condensation.

*Drosophila* oocytes rely on maternal RNAs and proteins produced in the adjacent, supporting nurse cells which are subsequently deposited into the oocyte [1,2,7,57]. To support egg development in the absence of transcription in the oocyte, many RNP granules are highly optimised to ensure long term storage and translational repression of maternal mRNAs until fertilisation [7,58–60]. Importantly, homologues and orthologues of *Drosophila* RNPs have been shown to phase separate in other systems, including yeast, *C.elegans* and zebrafish [15,61–63]. With many experimental advantages, *Drosophila* eggs offer a powerful system to investigate the physical principles and biological role of RNP condensation in early development.

In this perspective, we provide a brief overview of RNP granules in *Drosophila* oogenesis and highlight examples of liquid-like behaviour observed during early development. We then discuss how key concepts and models from LLPS could be used to understand the physical, structural and molecular principles regulating granule assembly and function during oogenesis. We conclude by highlighting how a multi-disciplinary approach using *in vitro* and *in vivo* studies, along with modelling, could better illustrate the physiological role of biomolecular condensates.

## Overview of maternal RNP granules in oogenesis

**Figure 1.**
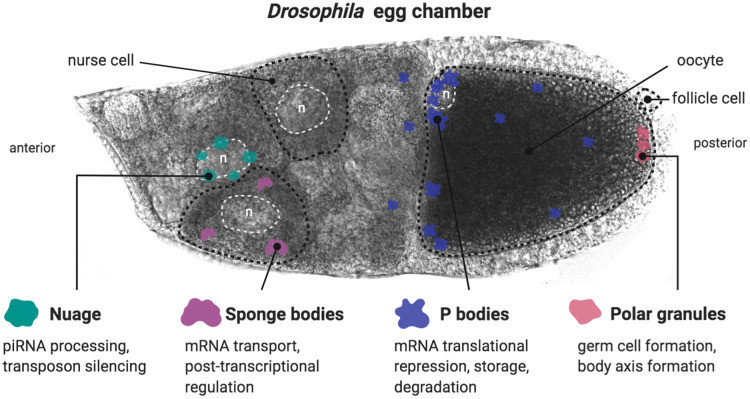
Schematic and role of maternal granules in egg chambers. Nuage is localised around the nurse cell nuclei, while sponge bodies are dispersed throughout the cytoplasm of the nurse cells. P bodies are enriched at the anterior margin of the oocyte (especially in the dorso-anterior corner). They are also observed throughout the oocyte and nurse cell cytoplasm. Polar granules are present at the posterior pole of the oocyte. Fifteen nurse cells, positioned to the anterior, produce the components (mRNAs, proteins, etc.) required for the development of a single oocyte. These germline-derived cells are interconnected through cytoskeletal bridges, allowing for cytoplasmic movement between them, and are surrounded by somatic-derived layer of follicle cells. (Representative cell types of the egg chamber are outlined with black dotted lines. Representative nuclei are outlined with white dotted lines and marked with an ‘n’). Created in BioRender.

Body axis patterning of *Drosophila* depends on the localisation, storage, translational control and degradation of maternal RNAs throughout oogenesis and early embryogenesis [1,2,58,64]. Several aspects of RNA metabolism during development are known to be regulated by membrane-less organelles, primarily RNP granules. Based on the presence of specific RNP components, both cytoplasmic (e.g. Balbiani bodies and U bodies) and nuclear granules (e.g. Cajal bodies, histone-locus bodies and induced nuclear bodies) have been described in *Drosophila* egg chambers [65–69]. As a comprehensive discussion of all RNPs identified in oogenesis is beyond the scope of this perspective, we summarise key similarities and differences among the well-studied cytoplasmic maternal RNP granules, namely; nuage, sponge bodies, processing bodies (P bodies) and polar granules (Figure 1). We acknowledge that the nomenclature in this field is not always consistent and that the contents of certain ‘bodies’ can be contentious. Here, we discuss the granules based on existing structural and compositional evidence.

## RNP granule similarities and differences

The first similarity is the absence of an outer membrane, thereby allowing RNP granules to rapidly and reversibly alter their composition in response to changes in cellular conditions such as pH, temperature and osmolarity. At egg activation, for example, a change in osmolarity is thought to cause P bodies in the mature oocyte to rapidly dissolve and release stored mRNAs for translation [69–71]. A second similarity is internal structuring within granules which creates an additional level of macromolecular organisation. Certain maternal mRNAs and RBPs for instance, are shown to be differentially partitioned within P bodies [62,69,72]. A third, obvious, similarity is that they all classify as RNP granules due to the presence of both proteins and RNAs. Therefore, physical principles underlying their biogenesis and regulation could be similar.

Despite these similarities, maternal RNP granules have unique functions at different stages of development. Nuage, the earliest visible RNP granules localised around nurse cell nuclei, are proposed to be sites of piwi-interacting RNA (piRNA) processing and transposon silencing while sponge bodies package and transport translationally repressed maternal mRNAs [65,73–76]. P bodies and polar granules are unique as their roles extend beyond oogenesis into embryogenesis. While P bodies help facilitate RNA storage and translational regulation, polar granules at the posterior of the oocyte function to sequester factors required for the formation of the embryonic germ cells [77–79]. A second key difference is compositional diversity among the different granules (Table 1). Importantly, granules such as sponge bodies, P bodies and polar granules, are not static in their protein composition, but rather are able to dramatically alter their composition to facilitate specific functions. Sponge bodies, for example, change their composition and dynamics immediately upon entry into the oocyte from the adjacent nurse cells [65]. Other notable differences such as size and morphology between maternal granules may exist, however, these features are less well characterised. It is likely that the morphological and compositional differences between granules dictate their material states. Even with these differences, structural and molecular similarities suggest that common underlying physical principles regulate the properties of maternal RNP granules.

**Table 1:** Compositional diversity and location of selected maternal RNP granules

Granule type	Proteins enriched	Location
Nuage	**Ago3**, Armitage, **Aubergine**, Krimper, Maelstrom, **Me31B**, Papi, Qin, Spindle-E, Squash, Tejas, **Trailer hitch**, **Tudor**, **Vasa**, Zucchini	nurse cells
Polar granules	**Aubergine**, **Dcp-1**, eiF4A, **Me31B**, **Oskar**, Piwi, Pyruvate kinase, 6-phosphofructokinase, **Staufen**, Ter 94, **Tudor**, **Vasa**	oocyte, embryo
P bodies	**Ago-3**, **Bruno**, **Cup**, **Dcp-1**, **Dcp-2**, Edc3, **eiF4E**, **Exuperentia**, Growl, Hpat, **Hrb27C**, **Me31B**, **Orb**, Pacman, **Staufen**, **Squid**, **Trailer hitch**	nurse cells, oocyte, embryo, adult neurons
Sponge bodies	BicC, **Bruno**, Btz, **Cup**, **Dcp-1**, **Dcp-2**, **eiF4E**, **Exuperentia**, Gus, **Hrb27C**, **Me31B**, **Orb**, **Oskar**, **Squid**, **Trailer hitch**	nurse cells, oocyte

Proteins shown to be enriched/localised in selected maternal granules. In bold are proteins associated with more than one granule. Whilst this is not an exhaustive list of proteins, those included are the most well-understood relative to each granule. With many shared proteins, it is important to consider testing a combination of different markers when studying RNP granules in development.

## Examples of phase separation during early *Drosophila* development

Despite sharing several proteins, how maternal RNP granules regulate their composition is a long-standing question. Our understanding of the biophysical and biochemical principles that govern granule diversity, assembly and disassembly has recently benefited from new conceptual frameworks. LLPS has emerged as an attractive model to explain the observed properties of membrane-less organelles, including RNP granules [11,48,80–83]. The earliest example of maternal RNP structures shown to exhibit liquid-like behaviour were induced ‘bodies’ found in the *Drosophila* oocyte nucleus. These bodies are highly dynamic, with frequent fusion events and exchange of molecules between the bodies and the nucleoplasm. Interestingly, their formation was induced by changes in the salt concentration, indicating that weak electrostatic interactions may govern their assembly [84,85].

Another example from oogenesis occurs in the cytoplasm when axis patterning maternal mRNAs, *bcd* and *oskar (osk)*, enter the oocyte at the anterior margin from the adjacent nurse cells and independently coalesce into larger particles. These separate RNP associations localise to opposite poles of the oocyte, where they are anchored [86,87]. While the biological importance of coalescence and the impact on granule properties is not clear, it is plausible that coalescence leads to increased interactions that stabilise over time, likely to assist in anchorage.

In the early embryo, a key nuclear protein associated with heterochromatin assembly and function, Heterochromatin Protein 1 alpha (HP1α), phase separates to form dynamic liquid-like individual heterochromatin modules that become less dynamic, more stable with time [88]. This phase transition is accompanied by changes in the morphology and material state of HP1α, likely enabling stronger DNA compaction. Similarly, polar granule components such as Osk protein, exists as phase separated compartments exhibiting liquid-like and hydrogel-like properties [89]. Together, these examples suggest that RNP liquid-like properties and LLPS are a common phenomenon in *Drosophila* development. This is an appealing prediction as RNP granules can be regulated by developmental cues and dynamic molecular interactions. Below we ascribe the current knowledge of condensate properties for investigating RNP granules to elucidate their physiological role in development.

## Compositional control

Establishing a condensed network of interacting macromolecules is an essential step in granule assembly [90]. According to the ‘scaffold and client’ model, scaffolds are essential proteins that help promote granule assembly, while clients are proteins that transiently interact with scaffolds and regulate condensate properties [91–93]. While this model has primarily been explored *in vitro*, RNP granules in the developing egg are a powerful *in vivo* system to test the model and have the advantage of overlapping RBPs associated with different granules. This is exemplified by the piRNA binding protein Aubergine (Aub), which behaves as a scaffold or client depending on the granule it is associated with. While *aub* mutants result in a partial loss of nuage in the nurse cells, these mutants completely disrupt polar granule formation at the posterior of the oocyte [94,95]. Identifying and testing scaffold and client proteins *in vivo* with genetics would be challenging since many RNPs are essential for egg chamber development in *Drosophila*. Therefore, reconstituting maternal granules *in vitro* through a minimal system of scaffold and client proteins, under physiological conditions, is an important alternative strategy [96]. This approach will provide insights into how RNP interactions regulate granule composition and enable systematic experimentation to identify the underlying sequence and structural determinants of scaffolding proteins.

## Material properties

RNP granules can exist in diverse material states, such as liquid, gel or solid, each of which has a distinct functional consequence [22,62,96–99]. Balbiani bodies, for example, exhibit solid-like material state likely facilitating stable storage of organelles and macromolecules during oocyte dormancy [61,96]. Material states of RNPs have been largely explored *in vitro*, but how these properties impact biological function is less well understood.

Mature *Drosophila* oocytes can be stored for multiple days without affecting RNA levels [100]. This efficient storage of RNAs is likely through RNP granules adopting a stable material state. P bodies are an example of storage sites for maternal mRNAs during oogenesis. However, P bodies are more complex as *grk* mRNA associated with P bodies is translated during mid-oogenesis while *bcd* mRNA is stored in P bodies until egg activation [69]. How P bodies change material states to perform different functions in development is key to understanding their role in translational regulation. One clue comes from experiments on Maternal expression at 31B (Me31B), a conserved RNA helicase found in many storage granules including *C. elegans* germ granules and mammalian somatic P bodies [62,66,101]. While knockdown of Me31B shows premature translation of stored mRNAs during early oogenesis [66], whether Me31B mutants affect P body material state remains unknown. However, it is exciting to consider that these mutant P bodies could have less stable material properties resulting in premature mRNA release and subsequent translation. Interestingly, P bodies from arrested *C. elegans* oocytes adopt a semi-liquid, viscoelastic material state which allows both stability and flexibility for RNA regulation [62]. Considering the similarities in P body components between these systems, we speculate that *Drosophila* P bodies would adopt a similar material state. Comprehensive characterisation of material properties using a combination of genetics and quantitative live imaging of RNP components will provide key insights into how granule physical states are regulated in response to cellular and developmental cues.

## Multilayered organisation

Although RNP condensates contain thousands of diverse macromolecules, for a long time they were considered homogenous in organisation. High resolution microscopy revealed that condensates can possess structured internal organisation on multiple scales. The nucleolus, for example, shows multiple liquid phases coexisting in the same granule giving rise to its heterogenous internal organisation [102,103]. While multi-phase organisation has also been reported in stress granules, P bodies, and P granules, the biological significance remains less clear [62,69,72,104].

Nuage during early *Drosophila* oogenesis exhibits levels of internal structuring with at least two sub-domains, one with Aub and another with Aub and Argonaute-3 [105]. Each internal level regulates a different step in the piRNA processing pathway in the nuage, supporting a model where proteins in different layers of an RNP granule can execute different functions. *Drosophila* P bodies are another example of granules shown to possess structured internal organisation, in this case a shell and core architecture is proposed. Specific mRNAs and RBPs are shown to be enriched in different layers of the P body, thereby facilitating differential translational regulation [69].

To resolve how different components contribute to the overall material state and function of RNP granules, a combination of super resolution imaging and quantitative single molecule assays should be used [106]. This would reveal finer details and localisation of specific molecules along with the material state of the RNP in question.

## The role of RNA

RNA storage and translational control is likely a major function of RNP granules in oogenesis as they form in response to high levels of untranslated mRNAs and disperse at a time when many mRNAs are translated. While proteins are typically considered to be the key scaffolds for granule assembly, more recently RNA has also been shown to both phase separate and drive the assembly of RNP condensates [80,83,107,108].

In the oocyte, certain localised mRNAs appear to coalesce into larger, less dynamic particles at their destination. This apparent change in the physical state is also accompanied by their association with RNP granules, such as P bodies or polar granules. Separate studies have also demonstrated that RNAse treatment results in the breakdown of RNP granules, highlighting the importance of RNA in maintaining the integrity of RNP granules [66,78].

These observations suggest a model whereby ‘sticky’ mRNAs promote granule nucleation by concentrating key scaffold proteins through sequence specific binding and subsequently regulate stability and material property. Testing this model *in vivo* requires developing techniques to selectively disrupt mRNAs while observing the effect on the RNP granules [80]. Complementarily, *in vitro* transcribed RNAs could be used in combination with specific scaffold proteins to reconstitute RNP granules. If successful, *in vitro* studies would be amenable to testing RNA sequences, for example in the untranslated regions, in the formation and regulation of RNP granules.

## Concluding remarks

**Figure 2.**
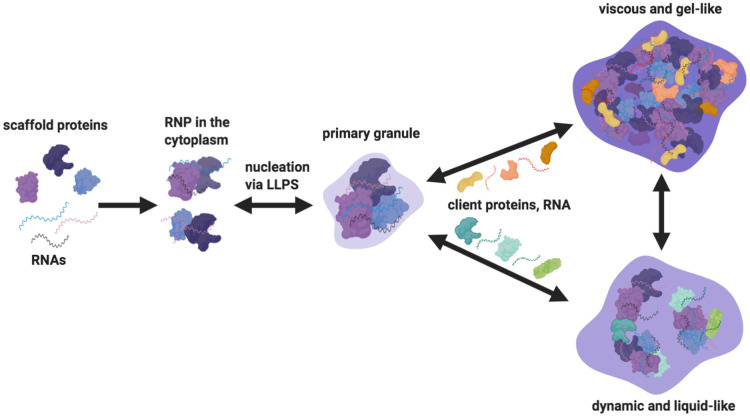
Model for RNP granule assembly and maturation in *Drosophila* egg chambers. In the cytoplasm, key scaffolding proteins and mRNAs, through multivalent interactions, come together to form RNP complexes. Multiple RNP complexes nucleate to assemble a primary granule via LLPS. Depending on the partitioning of specific client proteins and RNAs, granule diversity may be achieved. Although client-scaffold interactions may already be present during primary granule assembly, our model proposes that higher partitioning of clients regulate granule material states by modulating the strength of the resulting molecular interactions. While liquid-like and gel-like physical states are more commonly observed *in vivo*, other material states can exist based on specific developmental and environmental cues. Created in BioRender.

The field of phase separation has made clear progress towards deciphering the physicochemical rules governing biomolecular condensates. Since the majority of the data are derived from *in vitro* reconstitution and cell culture studies, there is still much to learn about the functional role of phase separation at a biological level. Here, we have highlighted how granule properties impact function and discussed the potential for *Drosophila* oogenesis to be used for investigating fundamental principles of phase separation in RNP granule assembly, organisation and material properties (Figure 2).

Fundamentally, it is extremely challenging to control all of the variables and factors that regulate condensates *in vivo*. Therefore, *in vitro* studies, including RNP granule purification, are important for identifying key features, such as sequence determinants, the role of non-equilibrium factors and multivalent RNP interactions [109,110]. Together, a combination of *in vitro* studies, modelling and *in vivo* assays will be required to fully comprehend the physiological functions of biomolecular condensates in cell and developmental biology.

## Summary

- Liquid–liquid phase separation is an emerging paradigm to understand biomolecular condensates and their roles in regulating cellular processes.
- RNP granules are highly conserved biomolecular condensates involved in regulating RNA metabolism.
- Diverse maternal mRNAs are regulated by RNP granules during *Drosophila* oogenesis.
- Studying the physicochemical principles of RNP granules in the *Drosophila* egg chamber could provide insights into the biological role of phase separation.

## Open Access

Open access for this article was enabled by the participation of University of Cambridge in an all-inclusive *Read & Publish* pilot with Portland Press and the Biochemical Society under a transformative agreement with JISC.

## Abbreviations

Aub: Aubergine
HP1α: heterochromatin protein 1 alpha
LLPS: liquid–liquid phase separation
Me31B: maternal expression at 31B
mRNAs: messenger RNAs
Osk: Oskar
P bodies: processing bodies
piRNA: piwi-interacting RNA
RBP: RNA binding proteins
RNP: ribonucleoprotein
